# Alignment-Free Machine Learning Serotype Classification of the Dengue Virus

**DOI:** 10.3390/v18030280

**Published:** 2026-02-25

**Authors:** Vladimir Gajdov, Isidora Prosic, Mihaela Kavran, Filip Bosilkov, Tamas Petrovic, Jelena Konstantinov, Gospava Lazic

**Affiliations:** 1 Scientific Veterinary Institute “Novi Sad”, 21000 Novi Sad, Serbia; vladimir.g@niv.ns.ac.rs (V.G.); tomy@niv.ns.ac.rs (T.P.); jelena.k@niv.ns.ac.rs (J.K.); goga@niv.ns.ac.rs (G.L.); 2 Faculty of Veterinary Medicine, University of Belgrade, 11000 Belgrade, Serbia; 3 Faculty of Agriculture, University of Novi Sad, 21000 Novi Sad, Serbia; 4 Faculty of Computer Science & Engineering, St Cyril and Methodius University, 1000 Skopje, North Macedonia; filip_b01@outlook.com

**Keywords:** k-mer composition, viral classification, vector-borne diseases, subtyping

## Abstract

Dengue virus (DENV) serotyping is essential for epidemiological surveillance, clinical risk assessment, and vaccine evaluation, as the four dengue serotypes differ in pathogenicity, immune interactions, and population dynamics. Existing subtyping methods largely rely on sequence alignment and phylogenetic inference, which can be computationally intensive and unreliable for short, fragmented, or error-prone sequences commonly generated in diagnostic and surveillance settings. There is a need for fast, alignment-free serotyping approaches that maintain high accuracy across heterogeneous sequence lengths while remaining scalable, transparent, and suitable for real-world diagnostic inputs. We demonstrate that compact 3-mer composition features are sufficient for highly accurate dengue virus serotyping when coupled with a lineage-aware Random Forest classification framework. Using 64 normalized 3-mer frequency features per sequence with ambiguity masking and enforcing strict cluster-aware validation at both 99% and 95% nucleotide identity thresholds, our approach achieved near-perfect accuracy and macro-F1 scores on held-out internal test sets. To further ensure independence, external validation datasets were filtered to remove exact sequence matches and any sequences sharing ≥99% or ≥95% nucleotide identity with internal data. On these strictly independent external datasets, the model maintained 100% accuracy and macro-F1 performance, confirming robust generalization beyond database redundancy. Robustness analyses showed stable performance under contiguous sequence truncation down to 300 bp and in the presence of ambiguous nucleotides, indicating resilience to realistic diagnostic inputs. These results demonstrate that a lightweight, alignment-free, machine learning approach can rival alignment-dependent methods while maintaining strict lineage-aware evaluation controls. The proposed framework combines high predictive accuracy, probabilistic reliability, computational efficiency, and reproducible validation design, making it well suited for large-scale genomic surveillance, rapid pre-screening, and diagnostic decision-support applications.

## 1. Introduction

Dengue fever is an acute infectious disease caused by the dengue virus, of the *Flavivirus* genus. Transmitted primarily by *Aedes* mosquitoes, this virus can lead to severe disease, potentially death, with around 400 million infections occurring per year [1]. Classification of viral subtypes is an elemental part of virus taxonomy within a virus species, with each subtype presenting a collection of genetic similarities among viruses from the total population. Virus subtypes can have considerable clinical significance in terms of their variation in pathogenesis, disease progression, susceptibility to drugs and vaccine development [2]. Presently, there are four subtypes of the dengue virus (DENV-1, DENV-2, DENV-3 and DENV-4) and infection with any one of these four subtypes provides only short-term, partial immunity (cross-protection) against the other three [3]. Moreover, the subtypes differ among themselves in terms of pathogenicity and virulence [4]. Therefore, it is crucial to identify the correct subtype/subtypes circulating in human and/or vector population and apply appropriate measures or therapy. The four serotypes include variable genotypes: six for DENV-1, two for DEN-2, five for DENV-3 and four for DENV-4 [5]. Classification systems for genotyping were initially based on greater than 6% pair-wise genetic distances within the genotype, using a 240-nucleotide sequence of the envelope (E)/nonstructural protein 1 (NS1) protein coding region [6]. Over time, as sequence data increased, these were replaced by systems based on entire protein-coding regions.

Until recently, most subtyping methods for the dengue virus have relied on alignment of an input sequence against a set of predefined subtype references sequences which enables algorithms to compute homologous sequence features or construct phylogenies. The Genome Detective [7], Dengue-GLUE [8], and Nextclade [9] tools are the main alignment-based frameworks for dengue virus subtyping. Genome Detective performs a two-step process combining BLAST-based serotype identification with maximum likelihood phylogenetic inference for lineage assignment. Dengue-GLUE relies on a Maximum Likelihood Clade Assignment (MLCA) and integrates query sequences into reference phylogenies to determine evolutionary placement. Nextclade classifies sequences based on unique nucleotide and amino acid substitutions that define established clades. Additional alignment-based tools, such as DEN-IM [10] and the Flavivirus Typing Tool (https://mpf.rivm.nl/mpf/typingtool/flavivirus/), enable genotyping from high-throughput sequencing data or across multiple flaviviruses, respectively, but offer lower resolution than lineage-specific methods like Dengue-GLUE and Genome Detective. Opposed to these alignment-dependent systems, CRAFT [11] introduces an alignment-free, machine learning-based framework for dengue virus subtyping that combines a Bitwise Chaos Game Representation (BCGR) feature extraction approach with a Random Forest classifier. The method supports hierarchical classification across serotype, genotype, and major and minor lineage levels, achieving 99.5% accuracy on a consensus hold-out test set and maintaining high performance even when classifying short sequence fragments of approximately 700 nucleotides. In this way, CRAFT eliminates the need for computationally intensive sequence alignment and phylogenetic inference and demonstrates that machine learning approaches can offer scalable, high-throughput alternatives for genomic classification of DENV.

Short oligonucleotide composition has been shown to differ across viral families and within viral species, reflecting underlying evolutionary constraints, mutational pressures, and host–virus interactions. Dinucleotide and trinucleotide usage patterns capture genome-wide biases shaped by replication mechanisms, immune evasion strategies, and long-term adaptation. Di Giallonardo et al. demonstrated that dinucleotide usage can cluster viruses by family more strongly than by host species, highlighting the phylogenetic signal embedded in short compositional motifs [12]. Fros et al. showed for Zika virus that CpG and UpA biases reflect competing selective pressures between vertebrate hosts and mosquito vectors [13], while He et al. reported lineage-specific dinucleotide biases in sugarcane mosaic virus [14]. More recently, alignment-free k-mer-based approaches have been successfully applied to viral classification and subtyping tasks, demonstrating that higher-order compositional features can retain discriminative power under strict independence constraints and across heterogeneous sequence lengths [15,16]). In this study, we introduce Fastvir (https://nivns.edu.rs/fastvir/), an alignment-free machine learning framework for dengue virus serotyping based on normalized 3-mer frequency features and a lineage-aware Random Forest classifier. By leveraging ambiguity-masked trinucleotide composition and strict cluster-aware validation at 99% and 95% nucleotide identity thresholds, Fastvir captures genome-wide compositional signatures sufficient for highly accurate and well-calibrated serotype prediction while avoiding redundancy-driven performance inflation.

## 2. Materials and Methods

### 2.1. Study Design and Objective

We developed and evaluated a machine-learning model to classify DENV serotypes (DENV-1–4) directly from nucleotide sequence data. The objective was to construct a simple, reproducible, and alignment-free workflow capable of operating across a wide range of sequence lengths (partial amplicons to near-complete genomes), using transparent compositional features robust to alignment or annotation differences, while producing deployable models with predictable generalization. Model performance was compared against alternative dengue virus subtyping approaches, including alignment-based and composition-based baselines.

### 2.2. Data Acquisition, Preprocessing, and Lineage-Aware Partitioning

All sequence data were retrieved from NCBI GenBank [17], comprising 40,081 DENV sequences across four serotypes (Table 1). Sequences shorter than 300 bp and exact duplicates were removed. Clustering was performed using MMseqs2 at 95% and 99% nucleotide identity (90% coverage threshold). No additional filters were applied, allowing the inclusion of sequences from any geographic location or collection date. After filtering, 36,658 sequences remained (DENV-1: 14,069; DENV-2: 12,186; DENV-3: 7011; DENV-4: 3392).

**Figure 1:**
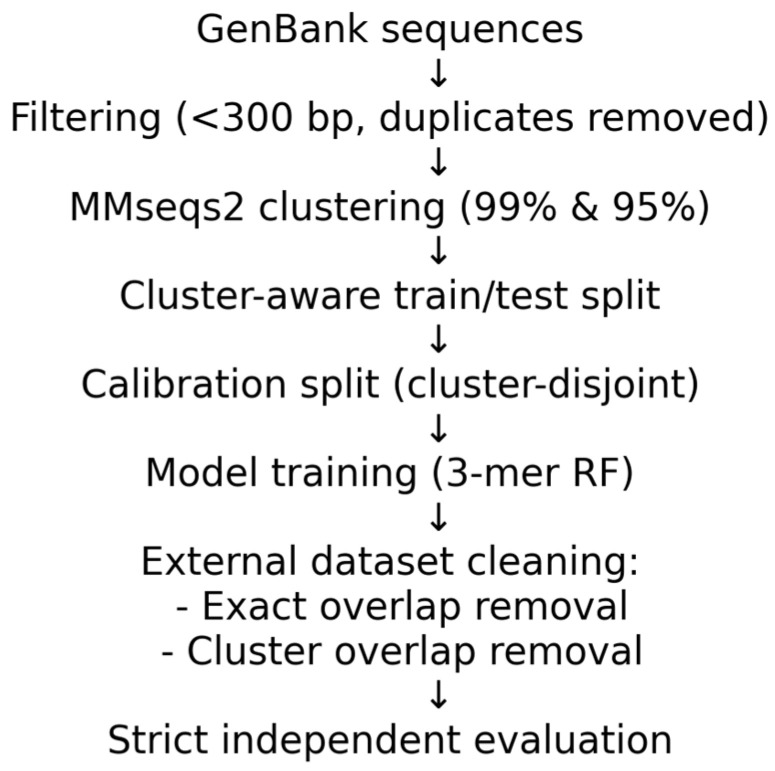
Lineage-aware evaluation framework. Schematic overview of the strict evaluation pipeline used in this study.

Cluster-aware train/test splits were constructed to ensure strict lineage independence. Multiple candidate splits were evaluated, and the partition minimizing deviation from the global serotype distribution was selected. Under strict 99% clustering, 6818 clusters were divided into 5454 training and 1364 test clusters (27,048 train; 9610 test sequences). Under 95% clustering, 1929 clusters were divided into 1543 training and 386 test clusters. Cluster membership was explicitly verified to ensure zero overlap between partitions. To quantify uncertainty while accounting for phylogenetic dependence, cluster-aware bootstrap resampling was performed (2000 replicates), treating clusters as the resampling unit and deriving 95% confidence intervals from percentile bounds. Sequence length distributions between training and test partitions were compared using two-sided Kolmogorov–Smirnov tests, with Benjamini–Hochberg FDR correction. Serotypes were flagged if KS statistic > 0.05 and adjusted *q*-value < 0.05. The lineage-aware evaluation framework is illustrated in Figure 1.

### 2.3. Feature Engineering and Compositional Analysis

Each sequence was transformed into a 64-dimensional vector of normalized 3-mer frequencies representing all ordered triplets in {A, C, G, T}^3^. Sequences were uppercased, and uracil (U) was converted to thymine (T). A sliding window (positions i–i + 2) enumerated 3-mers. Any triplet containing non-ACGT characters was excluded (k-mer-level ambiguity masking). Counts of valid 3-mers were normalized by total valid triplets, yielding relative frequency vectors. To assess GC-driven effects, a logistic regression baseline using only GC proportion was evaluated under identical cluster-aware splitting. Additionally, k-mer features were residualized against GC content via linear regression (training-derived coefficients applied to both partitions), removing linear GC-dependent variation while preserving higher-order compositional structure. SHAP (TreeSHAP) values were computed on held-out test sets under both clustering regimes. Global importance was defined as mean absolute SHAP value aggregated across samples and classes. Stability of feature rankings was assessed using Spearman correlation and Jaccard overlap of top 20 features. To evaluate feature dimensionality effects, models were trained with k = 2, 3, and 4 under identical strict-99 splits and hyperparameters.

### 2.4. Model Development, Calibration, and Evaluation Framework

All analyses were conducted in Python (v. 3.11), using NumPy (v. 2.2.0) [18], pandas (v. 2.2.3) [19], scikit-learn (v. 1.6.1) [20], Matplotlib (v. 3.9.3) [21], joblib (v. 1.4.2) (https://joblib.readthedocs.io), SHAP (v. 0.47.2) [22], MMseqs2 (v. 18.8) [23], BLAST (v. 2.16.0+) [24] and minimap (v. 2.30) [25]. Random seeds were fixed throughout (seed = 42). Three-mer frequency vectors were used without additional scaling or dimensionality reduction to preserve interpretability. A diverse panel of classifiers was evaluated under strict cluster-aware splitting: k-Nearest Neighbors, Support Vector Machines, Logistic Regression, Stochastic Gradient Descent, Decision Tree, Random Forest, AdaBoost, Gaussian Naïve Bayes, LDA/QDA, and Multilayer Perceptron. Based on performance, calibration behavior, stability, and computational efficiency, Random Forest was selected. Hyperparameter optimization was performed using RandomizedSearchCV (10-fold cluster-aware cross-validation). The final configuration employed: n_estimators = 500, max_features = “sqrt”, class_weight = “balanced_subsample”, bootstrap = False, fixed random_state, n_jobs = −1. Probability calibration used isotonic regression with cluster-disjoint calibration splits (85% fit/15% calibration). Calibration was evaluated using: Top-1 Expected Calibration Error (15 bins), Macro one-vs-rest Brier score, Reliability diagrams (10 uniform bins), and Cluster-aware bootstrap (300 replicates) was used for calibration uncertainty.

### 2.5. Robustness Analyses and External Validation

To test robustness to fragment length, contiguous subsequences (L = 150–2000 bp) were sampled under strict 99% clustering. Models were retrained and evaluated across multiple seeds. Performance (accuracy, macro-F1, ECE, and Brier) was summarized by mean ± SD and cluster-bootstrap confidence intervals (2000 replicates). Envelope (E) and NS1 regions were extracted externally via coordinate mapping. Original cluster identifiers were preserved. Models were trained and evaluated under identical lineage-aware constraints. An independent dataset was used (https://github.com/grubaughlab/DENV-genomics), accessed on 18 February 2026. Exact sequence overlap with internal data was removed. Joint clustering with internal sequences identified and excluded ≥99% and ≥95% identity overlaps, generating strictly independent external sets. Three-mer features were computed identically. Strict-99 and strict-95 models were evaluated using accuracy, macro-F1, ECE, and Brier score. Reliability diagrams and descriptive statistics were computed. Megablast was performed against strict-99 training sequences. Top 1 hits determined predictions. Performance metrics and cluster-aware bootstrap confidence intervals were computed. External evaluation used identical procedures. CRAFT was evaluated under identical strict-99 partitions. Training and external datasets were reformatted to meet CRAFT input requirements. Predictions were merged via accession identifiers and evaluated using accuracy, macro-F1, and confusion matrices.

### 2.6. Deployment Model, Computational Efficiency, and Reproducibility

For final deployment, strict-99 training and test partitions were merged. A cluster-aware 85%/15% split was used for model fitting and isotonic calibration. The calibrated model was retained as the deployment artifact. Disk footprint of serialized models was measured. Structural complexity was quantified via tree count, node counts, and maximum depths. Runtime benchmarking measured feature extraction and inference speed using repeated timing with warm-up iterations. Memory consumption was assessed via tracemalloc and optional RSS measurements. All benchmark metadata are included in the reproducibility package. The full workflow including clustering files, training partitions, external overlap removal logs, feature index, trained models, environment specifications, and benchmark metadata is provided in the accompanying reproducibility repository. All random seeds were fixed, and cluster-aware grouping was enforced throughout splitting and resampling procedures to prevent lineage leakage. These materials enable full reconstruction of all experiments and validation results.

## 3. Results

### 3.1. Dataset Composition and Cluster-Aware Partitioning

After filtering and clustering, the final internal dataset consisted of 36,658 sequences distributed across DENV-1 (14,069), DENV-2 (12,186), DENV-3 (7011), and DENV-4 (3392). Under the strict 99% MMseqs2 clustering regime, 6818 clusters were identified and partitioned into 5454 training clusters and 1364 test clusters, yielding 27,048 training sequences and 9610 test sequences. The strict 95% clustering regime resulted in 1929 total clusters, partitioned into 1543 training and 386 test clusters. Explicit verification confirmed zero cluster overlap between training and test partitions under both regimes. Within the strict 99% test set (n = 9610), 8197 sequences contained only ACGT characters, while 1413 sequences contained at least one ambiguous nucleotide. The fraction of ambiguous bases per sequence was low overall (mean 0.0037, median 0.0), with no sequence producing an all-zero feature vector after ambiguity masking.

### 3.2. k-mer Dimensionality Comparison

**Figure 2:**
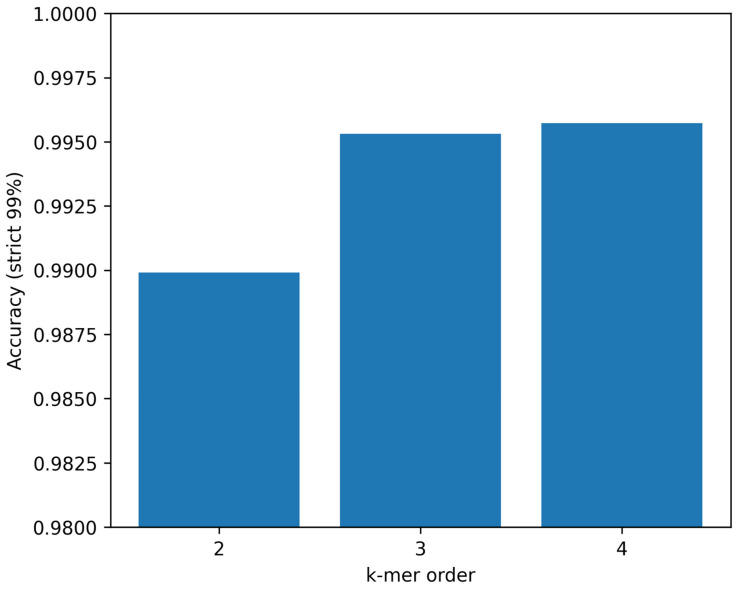
Performance vs. k-mer dimensionality. Comparison of Random Forest classification accuracy using 2-mer (16 features), 3-mer (64 features), and 4-mer (256 features) frequency representations under identical strict 99% cluster-aware splits.

Model performance improved with increasing k-mer dimensionality under identical strict 99% cluster-aware splits. Using 2-mers (16 features), the Random Forest achieved an accuracy of 0.9899 and macro-F1 of 0.9876. With 3-mers (64 features), accuracy increased to 0.9953 and macro-F1 to 0.9936. Using 4-mers (256 features), performance further increased slightly to 0.9957 accuracy and 0.9943 macro-F1. Given the modest performance gain from 3-mers to 4-mers relative to the 4× increase in feature dimensionality, the 3-mer representation was selected as the primary feature set. The relationship between k-mer dimensionality and classification performance is shown in Figure 2.

### 3.3. Performance of Baseline Classifiers

Sixteen classifiers spanning distance-based, margin-based, probabilistic, tree, and neural families were compared under 10-fold cross-validation with serotype × length-bin stratification. Eleven models performed well, but ensemble and neighborhood methods achieved near-perfect classification. The k-Nearest Neighbors (k = 10) reached 0.987 ± 0.0019 accuracy, closely followed by Random Forest (10 trees) at 0.986 ± 0.0018. The Multilayer Perceptron achieved 0.982, Decision Tree 0.974, and cubic SVM 0.976, while linear SVMs performed substantially worse (0.398) due to non-linear class boundaries. Training times varied from <0.02 s for kNN to ~0.5 s for Random Forest per fold.

### 3.4. Performance on Strict Cluster-Aware Internal Test Sets

**Figure 3:**
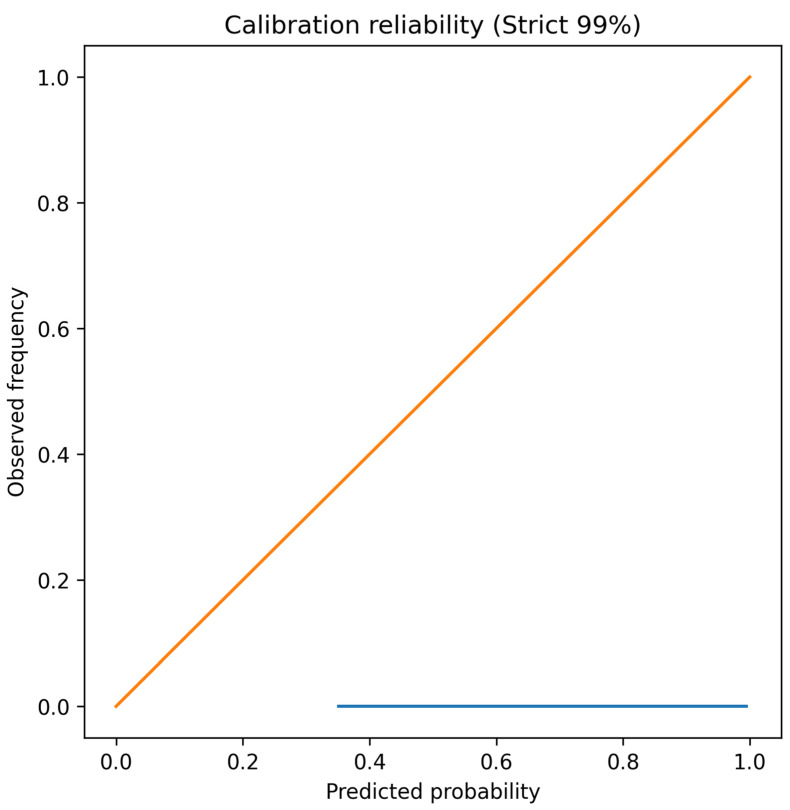
Reliability diagram showing predicted probabilities versus observed frequencies for the calibrated 3-mer Random Forest model.

Under the strict 99% cluster-aware test partition (n = 9610), the calibrated 3-mer Random Forest model achieved an accuracy of 0.99969 and macro-F1 of 0.99948 during the final deployment validation. On the calibration subset used for isotonic regression (n = 5602), uncalibrated performance was 0.99464 accuracy and 0.99176 macro-F1, which improved slightly after calibration to 0.99572 accuracy and 0.993 macro-F1. Performance was consistent across sequences with and without ambiguous bases. For pure ACGT sequences (n = 8197), accuracy was 0.99488 and macro-F1 was 0.99258. For sequences containing ambiguous bases (n = 1413), accuracy was 0.99788 and macro-F1 was 0.99821, indicating that k-mer-level ambiguity masking did not degrade predictive performance. The reliability of predicted probabilities is illustrated in Figure 3.

### 3.5. External Validation Under Strict Lineage Independence

The initial external dataset contained 13,540 sequences. Removal of exact sequence overlaps eliminated 2677 sequences. Additional removal of sequences sharing ≥99% identity clusters with internal data eliminated 10,513 sequences, yielding a strictly 99% cluster-independent external dataset of 350 sequences. Under the stricter 95% identity threshold, 10,743 sequences were removed, yielding 120 sequences. On the 99% cluster-independent external dataset (n = 350), the strict 99% trained model achieved 1.000 accuracy and 1.000 macro-F1. Calibration metrics were strong, with top-1 ECE of 0.02104 and macro one-vs-rest Brier score of 0.00125. On the 95% cluster-independent dataset (n = 120), accuracy and macro-F1 were also 1.000, with ECE of 0.04877 and macro Brier score of 0.00308. These results confirm robust generalization under strict identity-based independence constraints.

### 3.6. Cropping Experiment: Robustness to Partial Genome Input

**Figure 4:**
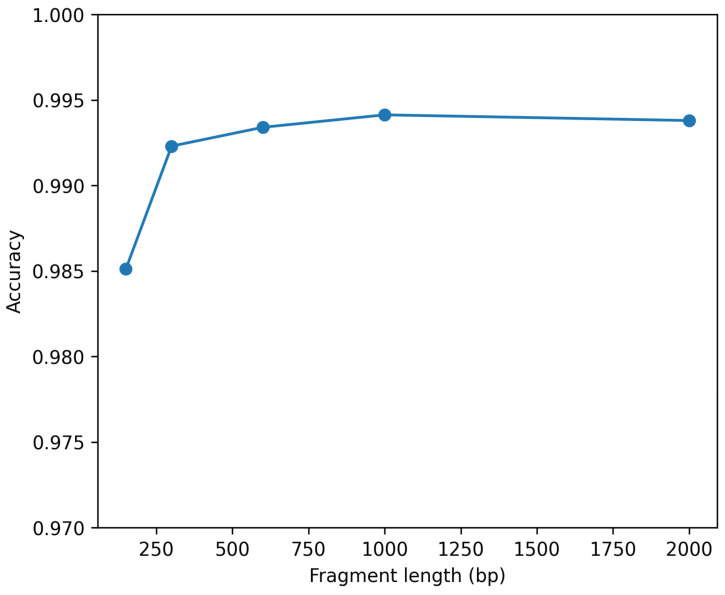
Robustness to partial genome input. Classification accuracy of the 3-mer Random Forest model as a function of contiguous fragment length (150–2000 bp) under strict 99% cluster-aware evaluation.

To assess robustness to partial sequences, contiguous subsequences of lengths 150, 300, 600, 1000, and 2000 nucleotides were sampled under strict 99% cluster-aware partitioning. Performance increased monotonically with fragment length. At 150 bp, mean accuracy across seeds was 0.9851 (SD 0.0020) with macro-F1 0.9796. At 300 bp, mean accuracy increased to 0.9923 and macro-F1 to 0.9897. At 600 bp, mean accuracy reached 0.9934 and macro-F1 0.9911. For 1000 bp and 2000 bp fragments, accuracy exceeded 0.9938 and macro-F1 exceeded 0.9922. Cluster-aware bootstrap confidence intervals confirmed stability. For example, at 600 bp, bootstrap mean accuracy was 0.9960 (95% CI: 0.9942–0.9973) and macro-F1 was 0.9947 (95% CI: 0.9926–0.9965). Calibration remained strong across fragment lengths, with ECE decreasing as fragment length increased. The relationship between fragment length and classification accuracy is shown in Figure 4.

### 3.7. Region-Restricted Modeling (E and NS1 Genes)

**Figure 5:**
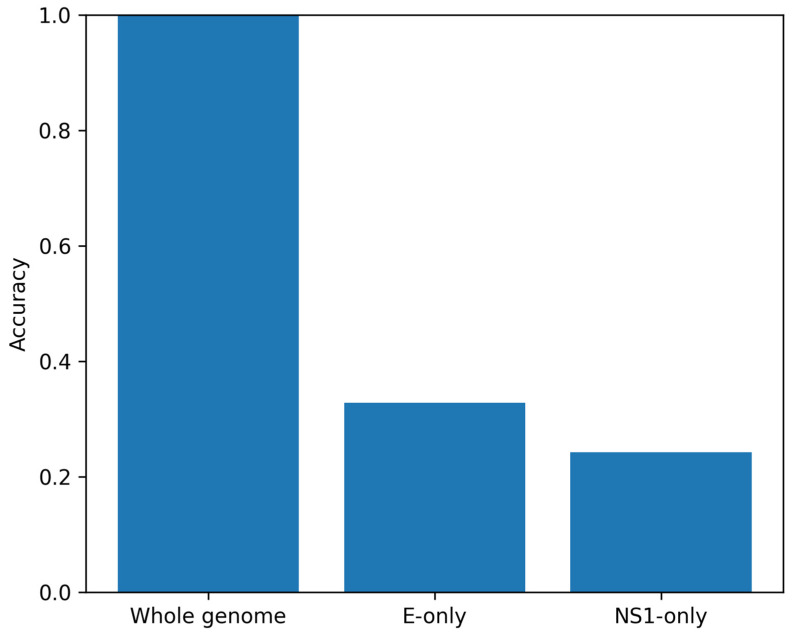
Region-restricted vs. whole-genome performance. Comparison of classification accuracy using whole-genome sequences versus region-restricted inputs (Envelope gene only and NS1 gene only).

When restricting analysis to the Envelope (E) gene only (n = 1163 test sequences), accuracy dropped to 0.3276 and macro-F1 to 0.1849, with ECE 0.2523 and macro Brier 0.2067. Under cluster-aware bootstrap resampling, mean accuracy was 0.3292 (95% CI: 0.2517–0.4028). Similarly, NS1-only modeling (n = 1139) yielded accuracy of 0.2423 and macro-F1 of 0.1862, with ECE 0.1715 and macro Brier 0.1931. Bootstrap mean accuracy was 0.2405 (95% CI: 0.1896–0.2836). These results indicate that discriminative compositional signal is not confined to individual genes but distributed genome-wide. Comparative performance between whole-genome and region-restricted models is presented in Figure 5.

### 3.8. Alignment-Based BLAST Baseline

Under strict 99% cluster-aware evaluation, BLAST achieved accuracy of 0.99781 and macro-F1 of 0.99764 on 9606 sequences with hits (four sequences had no hit), corresponding to a hit rate of 0.99958. Cluster-aware bootstrap estimates yielded mean accuracy 0.99779 (95% CI: 0.99667–0.99881) and macro-F1 0.99752 (95% CI: 0.99615–0.99872). On the 99% and 95% cluster-independent external datasets, BLAST achieved 1.000 accuracy and macro-F1, with 100% hit rates.

### 3.9. Composition-Based CRAFT Baseline

Under strict 99% evaluation, CRAFT achieved an accuracy of 0.99729 and macro-F1 of 0.99681 on the test set (n = 9610). On the 99% independent external dataset (n = 350), CRAFT achieved 1.000 accuracy and macro-F1. These results demonstrate that the 3-mer Random Forest model achieves performance comparable to or slightly exceeding composition-based baselines while retaining strict lineage-aware controls. A consolidated performance comparison across internal and cluster-independent external datasets is provided in Table 2.

### 3.10. Model Size and Computational Efficiency

The serialized calibrated deployment model had a disk size of 67,198,114 bytes (~67 MB). The base Random Forest model size was 67,195,289 bytes. The model consisted of 500 trees, with mean node count 1395.7 (median 1391; maximum 1567) and mean depth 28.5 (maximum 40). Feature extraction on 2000 sequences required a mean of 13.37 s (6.69 ms per sequence). Prediction required 0.134 s total (0.067 ms per sequence), and probability inference required 0.137 s (0.069 ms per sequence). Memory profiling showed peak Python allocation of approximately 6.19 MB during inference, indicating modest runtime memory requirements relative to full process memory.

## 4. Discussion

### 4.1. Methodological Rigor, Feature Design, and Model Validation

In this study, we developed and rigorously evaluated a compositional machine learning framework for dengue virus (DENV) serotype classification based on normalized 3-mer frequencies. Under strict 99% cluster-aware partitioning of 36,658 GenBank-derived sequences, the calibrated Random Forest classifier achieved an accuracy of 0.99969 and a macro-F1 score of 0.99948 on the held-out internal test set (n = 9610). Importantly, this evaluation was performed using lineage-aware grouping based on MMseqs2 clustering, ensuring that no sequences sharing ≥99% nucleotide identity were split across training and test partitions. Under the stricter 95% clustering regime, performance remained stable, demonstrating robustness to stronger independence constraints. External validation was conducted using an independently curated dataset subjected to both exact deduplication and identity-based cluster filtering against internal sequences. After removal of exact overlaps and ≥99% cluster overlaps, 350 sequences remained in the strictly independent 99% external dataset; at 95% identity independence, 120 sequences remained. On both datasets, the model achieved 1.000 accuracy and macro-F1, with well-controlled calibration metrics (ECE 0.021 and macro one-vs-rest Brier 0.0013 for the 99% independent set). These results indicate that performance is preserved under stringent lineage independence and external validation conditions. Calibration analysis demonstrated that predicted probabilities were reliable, with low Expected Calibration Error (ECE) and low macro Brier scores across internal and external evaluations. Calibration stability is particularly relevant for downstream decision-making in surveillance contexts, where probabilistic outputs may guide triage or quality control thresholds rather than binary decisions alone. A central concern in sequence-based machine learning is inflation of performance due to redundancy within public databases such as GenBank. This study explicitly addressed these issues at multiple levels. First, exact duplicate sequences were removed prior to analysis. Second, sequences were clustered at both 99% and 95% nucleotide identity using MMseqs2, and all model evaluations were conducted under cluster-aware splitting, ensuring that sequences within the same identity cluster were never divided between training and test sets. Explicit verification confirmed zero cluster overlap between partitions. Third, external validation datasets were subjected to both full-sequence string comparison to eliminate exact overlaps and joint clustering with internal data to remove any sequences sharing ≥99% or ≥95% identity with the internal dataset. Only cluster-independent sequences were retained for external testing. This multi-stage filtering ensures that the reported performance is not attributable to near-duplicate inflation or trivial memorization of highly similar sequences. The near-perfect classification performance observed under these constraints therefore cannot be explained by simple sequence redundancy. Instead, it reflects stable serotype-specific compositional signals that persist beyond close sequence identity and remain detectable under stringent lineage independence. We systematically compared 2-mer (16-dimensional), 3-mer (64-dimensional), and 4-mer (256-dimensional) frequency representations under identical strict 99% cluster-aware splits. While performance improved with increasing k-mer order, the marginal gain from 3-mers to 4-mers was small relative to the fourfold increase in dimensionality. Specifically, accuracy increased from 0.9953 (3-mer) to 0.9957 (4-mer), with comparable macro-F1 improvements. Given the trade-off between model complexity, interpretability, computational cost, and marginal performance gain, the 3-mer representation was selected as the primary model configuration. Biologically, k-mer frequencies capture serotype-specific compositional biases that arise from evolutionary divergence across the viral genome. Unlike alignment-based approaches, this representation does not rely on gene annotation or positional homology and therefore remains robust across partial sequences and genome rearrangements. The strong performance observed even under strict lineage-aware evaluation suggests that serotype discrimination is encoded in distributed compositional patterns rather than confined to specific diagnostic loci. Performance remained stable under the stricter 95% clustering regime, indicating that the model does not rely on extremely close sequence similarity for correct classification. External validation further confirmed that predictive accuracy is maintained when all sequences sharing high identity with internal data are removed. The model also demonstrated robustness to ambiguous nucleotide characters. Sequences containing non-ACGT bases were handled using k-mer-level masking rather than sequence-level exclusion. No test sequence produced an all-zero feature vector, and performance on sequences containing ambiguous bases was comparable to that on pure ACGT sequences. This indicates that ambiguity-safe feature extraction does not degrade classification performance. Cropping experiments showed that performance degrades gradually with decreasing fragment length but remains high even for relatively short contiguous regions. At 150 bp, mean accuracy remained above 0.98, increasing monotonically with fragment length. Calibration quality remained stable across fragment lengths, with decreasing ECE as sequence length increased. These results indicate that serotype-discriminative compositional signals are distributed genome-wide and detectable even in short fragments. In contrast, region-restricted modeling using only the Envelope (E) or NS1 gene resulted in substantially reduced performance, with accuracies of approximately 0.33 and 0.24, respectively. These findings further support the conclusion that serotype classification in this framework depends on genome-wide compositional structure rather than a single gene. Under strict 99% cluster-aware evaluation, BLAST achieved accuracy of 0.9978 and macro-F1 of 0.9976, slightly below the 3-mer Random Forest model. On cluster-independent external datasets, BLAST also achieved perfect classification among sequences with hits. CRAFT produced comparable results, with slightly lower internal accuracy but equivalent external performance. Conceptually, alignment-based methods such as BLAST rely on direct sequence similarity and remain appropriate for high-identity comparisons. In contrast, the compositional machine learning approach operates without alignment and provides calibrated probabilistic outputs. Runtime benchmarks demonstrated that inference is computationally lightweight, with prediction times on the order of 0.07 ms per sequence and modest memory requirements. This suggests potential advantages in large-scale or real-time screening contexts. The proposed approach is not intended to replace phylogenetic analysis but rather to provide a rapid, alignment-free classification layer suitable for surveillance pipelines, metagenomic preprocessing, or quality control.

### 4.2. Limitations, Practical Implications, and Future Directions

Despite rigorous controls, several limitations should be acknowledged. First, performance depends on the representativeness of the underlying database. Novel lineages with substantially altered compositional properties could challenge the classifier. Second, public database bias toward certain geographic regions or outbreak periods may influence feature distributions. Third, extremely short fragments or highly degraded sequences may reduce classification reliability, as observed in the cropping experiments at 150 bp. Finally, viral evolution may gradually shift compositional patterns, necessitating periodic retraining or incremental updates to maintain optimal performance. The compositional 3-mer Random Forest model provides a fast, lineage-aware, and calibration-stable approach to DENV serotype classification. Its independence from alignment and annotation makes it suitable for real-time surveillance, rapid triage of sequencing outputs, and integration into web-based diagnostic tools. The modest model size (~67 MB) and low inference latency support deployment in computationally constrained environments. Future work may extend this framework to genotype or lineage-level classification within serotypes, or to multi-virus classification across related flaviviruses. Comparative evaluation against deep learning architectures could further clarify the trade-offs between model complexity and interpretability. Finally, implementation of incremental updating strategies may allow continuous integration of newly deposited sequences while preserving strict lineage-aware validation principles.

## 5. Conclusions

We present a rigorously validated, cluster-aware compositional machine learning framework for dengue virus serotype classification that achieves highly accurate and well-calibrated predictions under strict lineage independence constraints. By combining exact deduplication, identity-based clustering at 95% and 99%, cluster-aware data splitting, and fully independent external validation, we demonstrate that performance is not driven by database redundancy or near-duplicate inflation. The selected 3-mer Random Forest model balances accuracy, computational efficiency, and interpretability, while remaining robust to ambiguous bases and partial genome input. Together, these results establish alignment-free compositional modeling as a reliable and computationally efficient complement to similarity-based methods for dengue serotype classification, with practical applicability to real-time genomic surveillance and large-scale screening workflows.

## Figures and Tables

**Table 1:** Number of DENV sequences before and after filtering.

Serotype	Before Filtering (*n*)	After Filtering (*n*)	Retained (%)
DENV-1	15,774	14,069	89.19
DENV-2	13,181	12,186	92.45
DENV-3	7377	7011	95.04
DENV-4	3749	3392	90.48
Total	40,081	36,658	91.46

**Table 2:** Performance comparison of the 3-mer Random Forest model, BLAST, and CRAFT on internal and cluster-independent external datasets.

Dataset	Model	Accuracy	Macro-F1
Internal strict 99% test (n = 9610)	3-mer RF (calibrated)	0.99969	0.99948
	BLAST (top-1)	0.99781	0.99764
	CRAFT	0.99729	0.99681
External 99% independent (n = 350)	3-mer RF (calibrated)	1.000	1.000
	BLAST	1.000	1.000
	CRAFT	1.000	1.000
External 95% independent (n = 120)	3-mer RF (calibrated)	1.000	1.000
	BLAST	1.000	1.000
	CRAFT	1.000	1.000

## Data Availability

The code used in this research is available upon request from authors.

## References

[1] Bhatt S., Gething P.W., Brady O.J., Messina J.P., Farlow A.W., Moyes C.L., Drake J.M., Brownstein J.S., Hoen A.G., Sankoh O. (2013). The global distribution and burden of dengue. Nature.

[2] Yung C.-F., Lee K.-S., Thein T.-L., Tan L.-K., Gan V.C., Wong J.G.X., Lye D.C., Ng L.-C., Leo Y.-S. (2015). Dengue Serotype-Specific Differences in Clinical Manifestation, Laboratory Parameters and Risk of Severe Disease in Adults, Singapore. Am. J. Trop. Med. Hyg..

[3] Rothman A.L. (2004). Dengue: Defining protective versus pathologic immunity. J. Clin. Investig..

[4] Bhatt P., Sabeena S.P., Varma M., Arunkumar G. (2020). Current understanding of the pathogenesis of dengue virus infection. Curr. Microbiol..

[5] Holmes E., Twiddy S. (2003). The origin, emergence and evolutionary genetics of dengue virus. Infect. Genet. Evol..

[6] Rico-Hesse R. (1990). Molecular evolution and distribution of dengue viruses type 1 and 2 in nature. Virology.

[7] Vilsker M., Moosa Y., Nooij S., Fonseca V., Ghysens Y., Dumon K., Pauwels R., Alcantara L.C., Vanden Eynden E., Vandamme A.-M. (2019). Genome Detective: An automated system for virus identification from high-throughput sequencing data. Bioinformatics.

[8] Hill V., Cleemput S., Pereira J.S., Gifford R.J., Fonseca V., Tegally H., Brito A.F., Ribeiro G., de Souza V.C., Brcko I.C. (2024). A new lineage nomenclature to aid genomic surveillance of dengue virus. PLoS Biol..

[9] Aksamentov I., Roemer C., Hodcroft E.B., Neher R.A. (2021). Nextclade: Clade assignment, mutation calling and quality control for viral genomes. J. Open Source Softw..

[10] Mendes C.I., Lizarazo E., Machado M.P., Silva D.N., Tami A., Ramirez M., Couto N., Rossen J.W.A., Carriço J.A. (2020). DEN-IM: Dengue virus genotyping from amplicon and shotgun metagenomic sequencing. Microb. Genom..

[11] van Zyl D.J., Dunaiski M., Tegally H., Baxter C., de Oliveira T., Xavier J.S. (2025). The INFORM Africa Research Study Group. Craft: A machine learning approach to dengue subtyping. Bioinform. Adv..

[12] Di Giallonardo F., Schlub T.E., Shi M., Holmes E.C. (2017). Dinucleotide Composition in Animal RNA Viruses Is Shaped More by Virus Family than by Host Species. J. Virol..

[13] Fros J.J., Visser I., Tang B., Yan K., Nakayama E., Visser T.M., Koenraadt C.J.M., van Oers M.M., Pijlman G.P., Suhrbier A. (2021). The dinucleotide composition of the Zika virus genome is shaped by conflicting evolutionary pressures in mammalian hosts and mosquito vectors. PLoS Biol..

[14] He Z., Qin L., Wang W., Ding S., Xu X., Zhang S. (2022). The dinucleotide composition of sugarcane mosaic virus is shaped more by protein coding regions than by host species. Infect. Genet. Evol..

[15] van Zyl D.J., Dunaiski M., Tegally H., Baxter C., de Oliveira T., Xavier J.S. (2025). Alignment-free viral sequence classification at scale. BMC Genom..

[16] Solis-Reyes S., Avino M., Poon A., Kari L. (2018). An open-source k-mer based machine learning tool for fast and accurate subtyping of HIV-1 genomes. PLoS ONE.

[17] Benson D.A., Cavanaugh M., Clark K., Karsch-Mizrachi I., Ostell J., Pruitt K.D., Sayers E.W. (2018). GenBank. Nucleic Acids Res..

[18] Harris C.R., Millman K.J., van der Walt S.J., Gommers R., Virtanen P., Cournapeau D., Wieser E., Taylor J., Berg S., Smith N.J. (2020). Array programming with NumPy. Nature.

[19] McKinney W. Data structures for statistical computing in Python. Proceedings of the 9th Python in Science Conference.

[20] Pedregosa F., Varoquaux G., Gramfort A., Michel V., Thirion B., Grisel O., Blondel M., Prettenhofer P., Weiss R., Dubourg V. (2011). Scikit-learn: Machine learning in Python. J. Mach. Learn. Res..

[21] Hunter J.D. (2007). Matplotlib: A 2D graphics environment. Comput. Sci. Eng..

[22] Lundberg S.M., Lee S.I. (2017). A unified approach to interpreting model predictions. Advances in Neural Information Processing Systems.

[23] Steinegger M., Söding J. (2017). MMseqs2 enables sensitive protein sequence searching for the analysis of massive data sets. Nat. Biotechnol..

[24] Camacho C., Coulouris G., Avagyan V., Ma N., Papadopoulos J., Bealer K., Madden T.L. (2009). BLAST+: Architecture and applications. BMC Bioinform..

[25] Li H. (2018). Minimap2: Pairwise alignment for nucleotide sequences. Bioinformatics.

